# Mitochondrial dysfunction is associated with hypertrophic cardiomyopathy in Pompe disease‐specific induced pluripotent stem cell‐derived cardiomyocytes

**DOI:** 10.1111/cpr.13573

**Published:** 2023-11-02

**Authors:** Wenjun Huang, Rui Zhou, Congshan Jiang, Jie Wang, Yafei Zhou, Xiaoyan Xu, Tao Wang, Anmao Li, Yanmin Zhang

**Affiliations:** ^1^ National Regional Children's Medical Center (Northwest), Key Laboratory of Precision Medicine to Pediatric Diseases of Shaanxi Province, Xi'an Key Laboratory of Children's Health and Diseases Shaanxi Institute for Pediatric Diseases, Xi'an Children's Hospital, Affiliated Children's Hospital of Xi'an Jiaotong University Xi'an China; ^2^ Department of Cardiology Xi'an Children's Hospital, Affiliated Children's Hospital of Xi'an Jiaotong University Xi'an China

## Abstract

Pompe disease (PD) is a rare autosomal recessive disorder that presents with progressive hypertrophic cardiomyopathy. However, the detailed mechanism remains clarified. Herein, PD patient‐specific induced pluripotent stem cells were differentiated into cardiomyocytes (PD‐iCMs) that exhibited cardiomyopathic features of PD, including decreased acid alpha‐glucosidase activity, lysosomal glycogen accumulation and hypertrophy. The defective mitochondria were involved in the cardiac pathology as shown by the significantly decreased number of mitochondria and impaired respiratory function and ATP production in PD‐iCMs, which was partially due to elevated levels of intracellular reactive oxygen species produced from depolarized mitochondria. Further analysis showed that impaired fusion and autophagy of mitochondria and declined expression of mitochondrial complexes underlies the mechanism of dysfunctional mitochondria. This was alleviated by supplementation with recombinant human acid alpha‐glucosidase that improved the mitochondrial function and concomitantly mitigated the cardiac pathology. Therefore, this study suggests that defective mitochondria underlie the pathogenesis of cardiomyopathy in patients with PD.

## Graphical abstract

PD patient's cardiac pathology was successfully recapitulated in PD patient‐specific iPSC‐derived cardiomyocytes, as evidenced by lysosomal accumulation of glycogen and hypertrophic cardiomyocytes. Defective mitochondria were associated with hypertrophic cardiomyopathy. Impaired fusion and autophagy of mitochondria and declined expression of mitochondrial complexes underlies the mechanism of dysfunctional mitochondria.

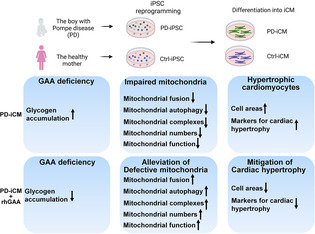

## 1 INTRODUCTION

Pompe disease (PD), which is also known as glycogen storage disease type II, is a rare autosomal recessive genetic disorder caused by the deficiency of acid alpha‐glucosidase (GAA).^1^, ^2^ GAA is localized in lysosomes and degrades lysosomal‐bound glycogen. Deficiency of GAA causes lysosomal glycogen accumulation in different tissues, and especially in cardiomyocytes, causing cardiac hypertrophy and eventually heart failure.^3^ PD can be classified into two forms, based on the age of onset and severity: classic infantile‐onset (or early‐onset) and nonclassic late‐onset. The classic form of infantile‐onset PD (IOPD) begins within a few months of birth and manifests as progressive skeletal muscle hypotonia and cardiac hypertrophy, and subsequently develops into heart failure between 3 and 5 months of age. Most patients would die by the age of 18 months without effective treatment.^3^, ^4^, ^5^ Although the glycogen‐induced lysosome damage was once considered the major molecular mechanism in the pathogenesis of PD, but accumulating evidence supports that the mitochondrial impairment induced by the aberrant glycogen accumulation also significantly contributes to the pathogenesis of PD.

Mitochondria are the central pivots for cellular metabolism and powerhouse for energy production.^6^ Perturbed metabolic signalling and mitochondrial dysfunction are common pathogenic mechanisms in patients with hypertrophic cardiomyopathy.^7^ Mitochondrial damage disrupts ATP synthase activity and ATP production by lowering the inner membrane potential to cause energy starvation and contractile failure in muscles.^8^ Patients with IOPD often present with signs of cardiac hypertrophy, and heart failure is a main cause of death in most patients.^9^ Recently, multiple mitochondrial defects have been described in induced pluripotent stem cells (iPSCs) derived from patients with PD and in the skeletal muscles in PD mouse models.^10^ However, it is currently unknown whether mitochondrial dysfunction precedes the development of heart failure, and if so, whether it promotes the transition from compensated cardiac hypertrophy to heart failure in patients with IOPD.

Herein, iPSCs from a patient with IOPD were used to explore the mechanism of cardiac hypertrophy in IOPD. Impaired mitochondrial functions including decreased mitochondrial biogenesis, oxygen consumption and ATP production were involved in the cardiac pathology in iPSC‐derived cardiomyocytes (iCMs) from a patient with IOPD. Mechanistic analysis indicated that impaired fusion and autophagy of mitochondria and declined expression of mitochondrial complexes underlies the mechanism of dysfunctional mitochondria. This was alleviated by the supplementation with recombinant human acid alpha‐glucosidase (rhGAA), which improved mitochondrial function and concomitantly mitigated cardiac pathology. Our discoveries support the newly proposed theory that mitochondria dysfunction is associated with hypertrophic cardiomyopathy in IOPD. Therefore, targeting the mitochondrial may serve as a novel candidate adjuvant therapeutic strategy in future clinical practice with PD.

## 2 MATERIALS AND METHODS

### 2.1 Subjects

A five‐month‐old child with PD and their mother were enrolled in the study. The investigation was conducted according the principles outlined by the declaration of Helsinki and was approved by the Ethics Committee of Xi'an Children's Hospital (No. 20210062), Affiliated Children's Hospital of Xi'an Jiaotong University. Written informed consents were obtained from the mother before the study.

### 2.2 Reagents

See Supporting Information for more details.

### 2.3 iPSC reprogramming and culture

See Supporting Information for more details.

### 2.4 Karyotype analysis

See Supporting Information for more details.^11^

### 2.5 Differentiation of three germ layers in vitro

See Supporting Information for more details.

### 2.6 Cardiomyocytes differentiation from iPSC

See Supporting Information for more details.^12^, ^13^

### 2.7 RNA isolation and qPCR

See Supporting Information for more details.

### 2.8 Immunofluorescence

See Supporting Information for more details.

### 2.9 Western blot assay

See Supporting Information for more details.

### 2.10 GAA activity assay

See Supporting Information for more details.

### 2.11 Periodic–acid–Schiff (PAS) staining

See Supporting Information for more details.^14^

### 2.12 Transmission electron microscopy

See Supporting Information for more details.

### 2.13 Seahorse assay

See Supporting Information for more details.

### 2.14 Reactive oxygen species (ROS) analysis

See Supporting Information for more details.

### 2.15 Mitochondrial membrane potential assay

See Supporting Information for more details.

### 2.16 Statistical analysis

Statistical analyses were performed using GraphPad Prism 9. Comparisons between two groups were analysed using Student's *t*‐test, and analysis of over two groups were performed using ANOVA. Data are presented as ‘mean ± SD’, with *p* < 0.05 considered as significant.

## 3 RESULTS

### 3.1 Characteristics of patient with IOPD and generation of iPSCs and iCMs

We previously reported a family with PD, where the five‐month‐old child with PD carried compound heterozygous mutations of c.1822C>T, p.R608X and c.2662G>T, p.E888X in the GAA gene inherited from both the father and mother, respectively^15^, ^16^, ^17^ (Figure S1A). Serum GAA activity of the proband was markedly reduced (2.1 nmol/(g min) vs. 24.8–93.3 nmol/(g min), proband vs. normal range, respectively). Ventricular hypertrophy was confirmed by echocardiogram and showed increased thickness of the free walls of the left and right ventricles (Figure S1C) and the septum. Electrocardiograph showed an ST‐T wave change and a biventricle hypertrophy pattern (Figure S1D). Serum cTnT and pro‐BNP levels were also elevated. Finally, mutations of c.2662G>T, p.E888X and c.1822C>T, p.R608X in the GAA gene in the family were confirmed with whole exon sequencing and Sanger sequencing (Figure S1B), supporting the diagnosis of PD.

The iPSCs from the proband (PD‐iPSCs) were previously generated and characterized in our laboratory.^15^, ^16^ To explore the molecular mechanisms underlying the cardiac hypertrophy observed in the proband, control iPSCs (Ctrl‐iPSCs) from the proband's mother who is phenotypically normal were reprogrammed from peripheral blood monocytes using the Sendai virus method. The Ctrl‐iPSCs showed the typical morphology of iPSC colonies (Figure S2A) with a normal karyotype via Giemsa‐banding (Figure S2C), and the heterozygous mutation of c.2662G>T, p.E888X in the GAA gene by Sanger sequencing (Figure S2B). Immunofluorescence (IF) results showed high expression levels of pluripotency markers, OCT4, SOX2, NANOG and SSEA4 (Figure S2D), and self‐renewal maker, Ki67 (Figure S2E). Furthermore, the *capacity* to *differentiate* into derivatives of all three germ layers was further confirmed by the trilineage differentiation assay (Figure S2F). These data suggest the successful establishment of the Ctrl‐iPSC.

Successfully differentiation of cardiomyocytes from iPSCs was shown via IF with increased expression of cTnT (Figure S3A), via qPCR with dramatically upregulated expression of ACTN2 and TNNT2 genes (Figure S3B), and via flow cytometry with nearly 100% of cells being positive for α‐actinin and cTnT (Figure S3C,D). The cell maturity did not significantly differ between the two groups as indicated by the expression of the maturity marker genes encoding myofibril (Figure S3E), calcium handling (Figure S3F) and ion channels (Figure S3G) at day 30 after differentiation.

### 3.2 Lysosomal glycogen accumulation in PD‐iPSC

**FIGURE 1:**
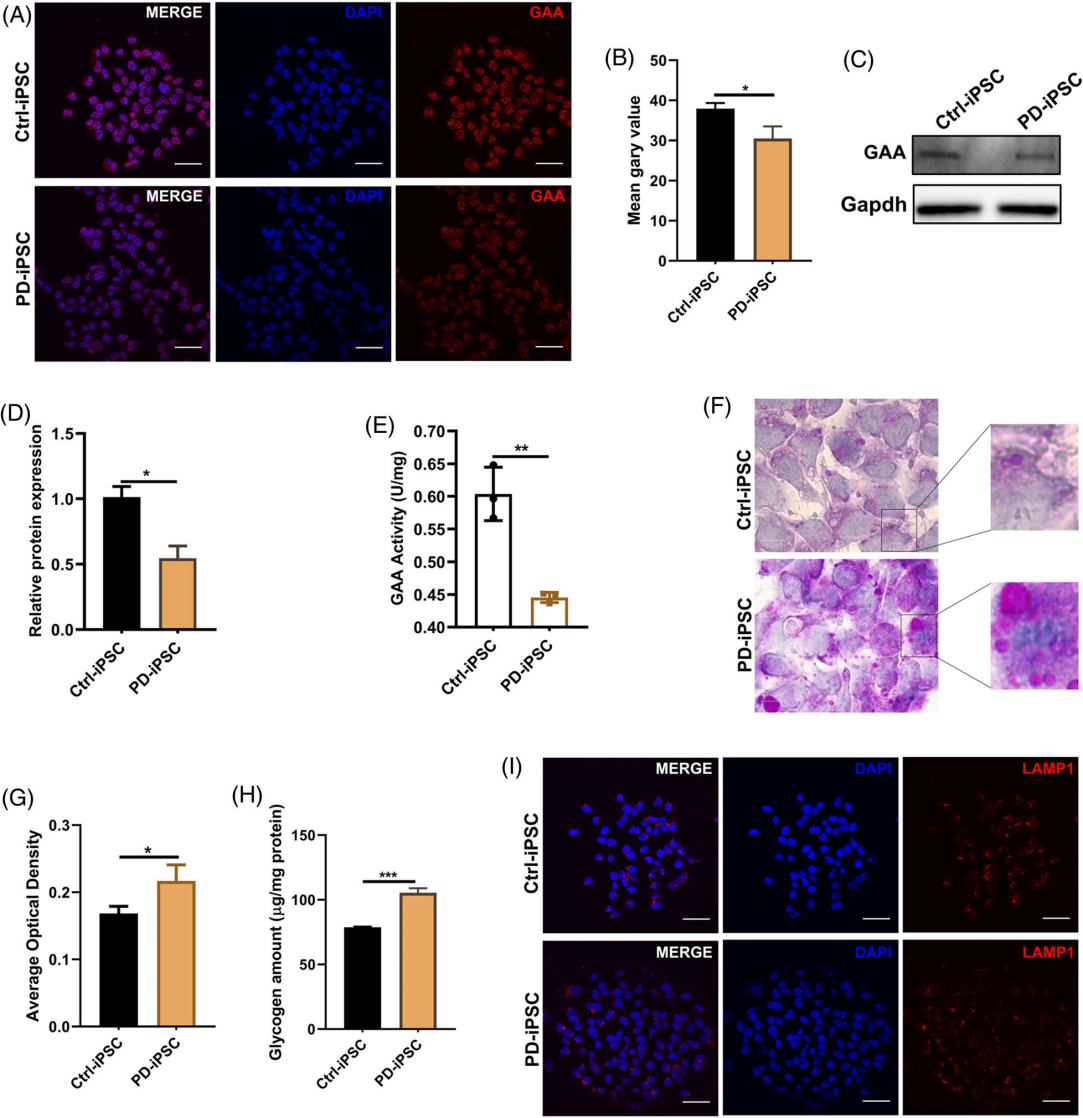
Lysosomal glycogen accumulation in iPSCs derived from a patient with PD (A,B) Representative images of immunostaining results (A) and quantitative data of fluorescent intensity (B) for GAA from PD‐iPSCs and Ctrl‐iPSCs; scale bar, 50 μm. Data are presented as the mean ± SD (number of random fields *n* = 5), *p < 0.05 (Student's *t*‐test). (C,D) Representative images of Western blotting results (C) and corresponding quantitative data of gray density (D) for GAA from PD‐iPSCs and Ctrl‐iPSCs. Data are presented as the mean ± SD (number of independent repeats *n* = 3) *p < 0.05 (Student's t‐test). (E) Lysosomal GAA activity of PD‐iPSCs and Ctrl‐iPSCs. All measurements were normalized to protein concentration and presented as the mean ± SD, **p < 0.01 (Student's *t*‐test). (F,G) Representative images of PAS staining of glycogen (F) and quantitative data of optical density (G) from PD‐iPSCs and Ctrl‐iPSCs; scale bar, 50 μm. Data are presented as the mean ± SD (number of random fields *n* = 5), *p < 0.05 (Student's *t*‐test). (H) Measurement of glycogen content from PD‐iPSCs and Ctrl‐iPSCs. All measurements were normalized to protein concentration and presented as the mean ± SD, ***p < 0.001 (Student's *t*‐test). (I) Representative images of immunostaining of LAMP1 from PD‐iPSC and Ctrl‐iPSC; scale bar, 50 μm. Three duplicates and three independent repeats were performed per experiment.

To investigate whether PD‐iPSCs exhibit the typical features of glycogen accumulation in lysosomes, the expression, activity and function of GAA were evaluated. IF results showed that the mean fluorescent intensity of GAA protein was much weaker in PD‐iPSC than Ctrl‐iPSCs (Figure 1A,B), which was confirmed via Western blotting (Figure 1C,D). In agreement, the GAA activity was markedly decreased in PD‐iPSCs compared with that in Ctrl‐iPSCs (Figure 1E), producing an increased accumulation of glycogen in lysosomes of PD‐iPSCs as indicated by the PAS staining results (Figure 1F,G) and quantitative detection of the amount of glycogen (Figure 1H). To exclude the possibility that lysosomes differed between the PD‐iPSCs and Ctrl‐iPSCs, the expression of lysosomal associated membrane protein 1 (Lamp1), a lysosome marker was tested using IF and showed that the mean fluorescence intensity was comparable between the two groups (Figure 1I). Thus, the compound mutations of the GAA gene are most likely responsible for the increased lysosomal glycogen accumulation in cardiomyocytes in the proband.

### 3.3 PD‐specific phenotype of the iCMs

**FIGURE 2:**
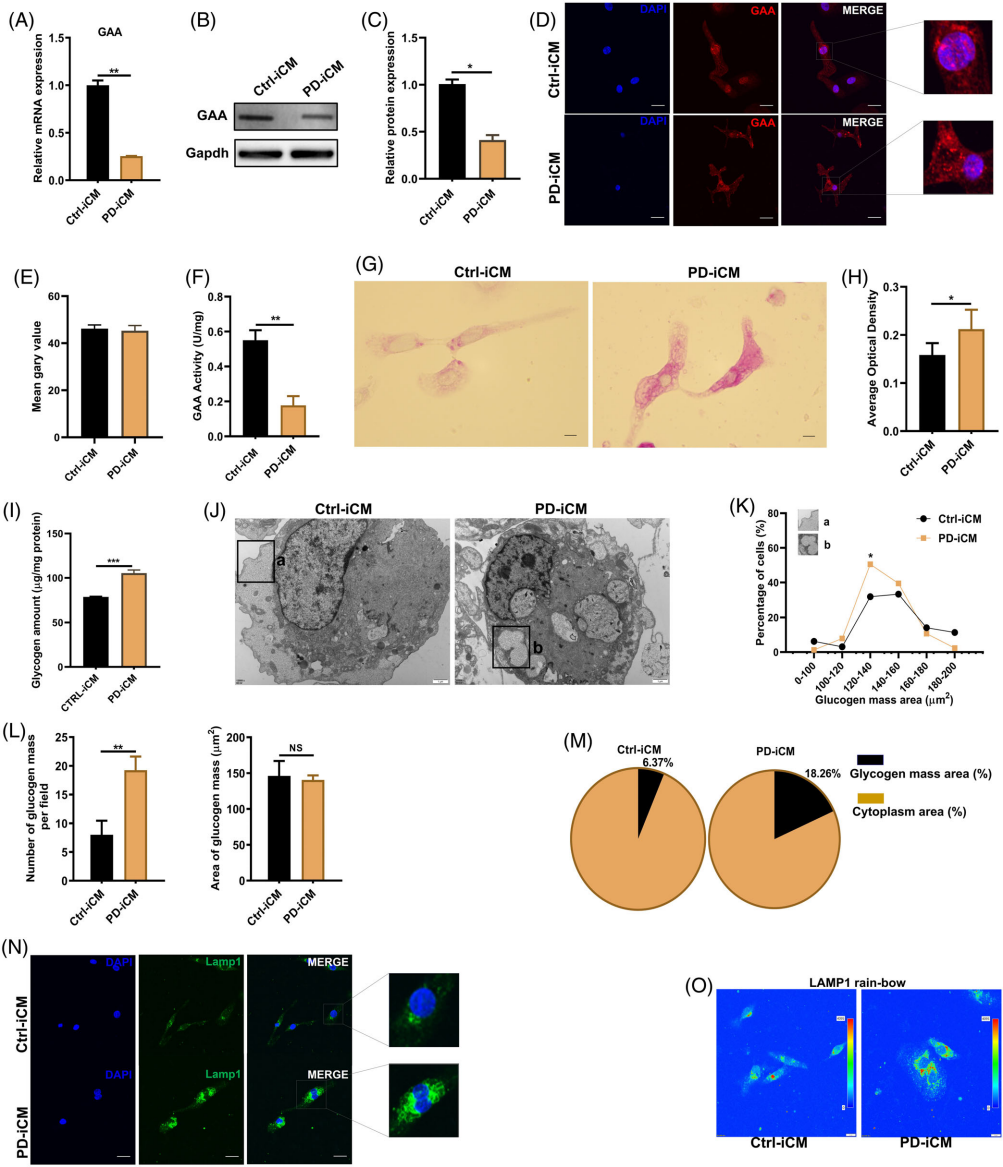
Glycogen accumulation in PD‐iCMs. (A) RT‐qPCR analysis of GAA mRNA expression in Ctrl‐iCMs and PD‐iCMs. Data are presented as mean ± SD with three duplicates. **p < 0.01 (Student's *t*‐test). (B,C) Representative images of Western blotting results (B) and corresponding quantitative data of gray density (C) for GAA from PD‐iCMs and Ctrl‐iCMs. Data are presented as the mean ± SD (number of independent repeats n = 3) *p < 0.05 (Student's t‐test). (D,E) Representative IF images of GAA (D) and quantification of the fluorescence intensity (E) from Ctrl‐iCMs and PD‐iCMs; scale bar, 50 μm. Data are presented as the mean ± SD (number of random fields *n* = 5) with Student's *t*‐test. (F) Comparison of GAA activity between Ctrl‐iCMs and PD‐iCMs. All measurements were normalized to protein concentration and presented as the mean ± SD, **p < 0.05 (Student's *t*‐test). (G,H) Representative images of PAS staining for glycogen (G) and quantitative data of optical density (H); scale bar, 50 μm. Data are presented as the mean ± SD (number of random fields *n* = 5) with three duplicates (Student's *t*‐test). (I) Measurement of glycogen content from PD‐iCMs and Ctrl‐iCMs. All measurements were normalized to protein concentration and presented as the mean ± SD; ***p < 0.001 (Student's *t*‐test). (J‐M) Representative TEM images (J) showing glycogen accumulation in Ctrl‐iCMs and PD‐iCMs. Glycogen accumulation was evaluated using the ImageJ software. Quantification of the percentage of cells in different glycogen mass areas (K), the number of glycogen mass and the area of glycogen mass (L) and the area ratio of glycogen mass relative to the whole cell (M). (N,O) Representative IF images of lysosomal marker, LAMP1 (N) and corresponding rainbow analysis of IF result (O) from Ctrl‐iCMs and PD‐iCMs. Three duplicates and three independent repeats were performed per experiment.

The heart is one of the most affected organs by PD, we consequently evaluated glycogen accumulation in the PD‐iPSC‐derived cardiomyocytes (PD‐iCMs). qPCR (Figure 2A) and Western blotting (Figure 2B,C) demonstrated that the expression of GAA was decreased at both mRNA and protein levels in PD‐iCMs compared with that in Ctrl‐iCMs. Representative IF results showed that the PD‐iCMs contained more coarse GAA granules than Ctrl‐iCMs (Figure 2D), although this do not significantly affect the intensity of the GAA signalling (Figure 2E). In line with our prediction, GAA enzyme activity was significantly weaker in PD‐iCMs than that in Ctrl‐iCMs (Figure 2F), producing an increased accumulation of glycogen in lysosomes of PD‐iCMs as indicated by the results of PAS staining (Figure 2G,H) and quantitative detection of the amount of glycogen (Figure 2I). Transmission electron microscopy^18^ was performed to further verify the glycogen accumulation in the lysosome compartment (Figure 2J–M). Increased numbers of glycogen particles were present in PD‐iCMs compared with those in Ctrl‐iCMs (Figure 2J). In particular, the percentage of cells containing the particles with a glycogen mass area of 120–140 μm^2^ (Figure 2K) was significantly increased in PD‐iCMs compared with that in Ctrl–iCMs (18.26% vs. 6.37%), respectively. Further analysis showed that the average number of glycogen mass per visual field was significantly higher in PD‐iCMs than that in Ctrl‐iCMs (Figure 2L). The average glycogen mass area was comparable (Figure 2L), but the ratio of the glycogen mass area relative to the whole cell was higher in PD‐iCMs than that in Ctrl‐iCMs (Figure 2M). Consistently, IF staining of LAMP1 showed that the GAA granules were much coarser in PD‐iCMs than that in Ctrl‐iCMs, indicating that the aggregation and swelling of lysosomes in PD‐iCMs was caused by over‐accumulated glycogen (Figure 2N,O).

### 3.4 The defective mitochondria were involved in progression of cardiac hypertrophy in PD‐iCMs

**FIGURE 3:**
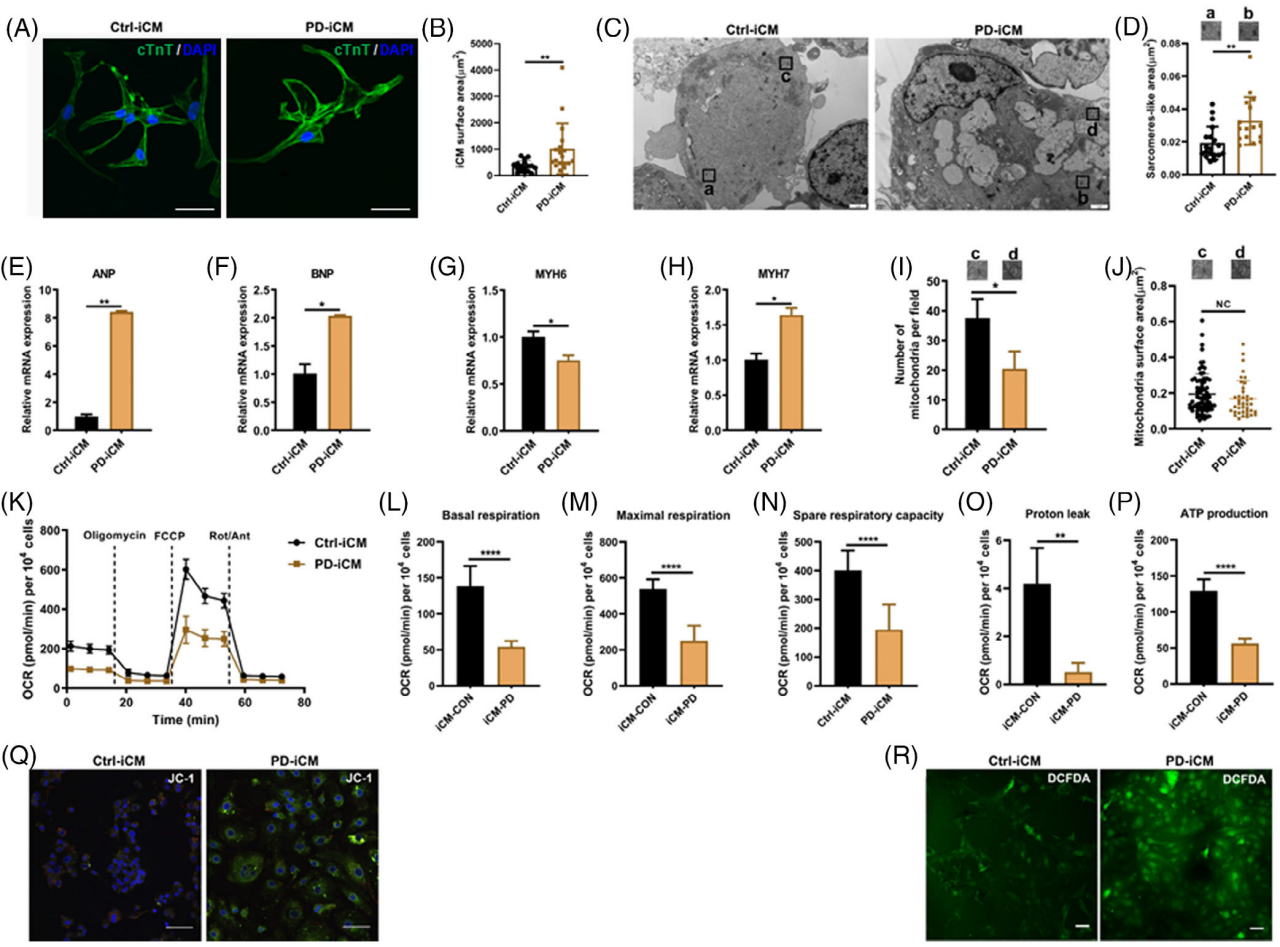
Phenotypes of cardiac hypertrophy in patients with PD was duplicated in PD‐iCMs. (A,B) Representative IF images of the expression of cardiomyocyte marker (cTnT) from Ctrl‐iCMs and PD‐iCMs (A). Quantitation of the average cell area; scale bars, 20 μm (B). Data are presented as the mean ± SD (cells number Ctrl‐iCMs *n* = 17, PD‐iCMs *n* = 18) with eight duplicates; **p < 0.01 (Student's *t*‐test). (C,D) Representative images of ultrastructure from Ctrl‐iCMs and PD‐iCMs. Enlarged images (a,b) indicating the sarcomere structure; scale bars, 1 μm (C). Sarcomere area was compared between Ctrl‐iCMs and PD‐iCMs (D). Data are presented as the mean ± SD (cell number: Ctrl‐iCMs *n* = 19, PD‐iCMs *n* = 16) with four duplicates; **p < 0.01 (Student's *t*‐test). (E–H) RT‐qPCR analysis for the mRNA expression of cardiac hypertrophy markers (ANP, BNP, MYH6 and MYH7) in Ctrl‐iCMs and PD‐iCMs. Data are presented as the mean ± SD with three duplicates; *p < 0.05 and **p < 0.01, (Student's *t*‐test). (I,J) TEM images showing the number (I) and area (J) of mitochondria in Ctrl‐iCMs and PD‐iCMs. Data are presented as the mean ± SD (cell number: Ctrl‐iCMs *n* = 75, PD‐iCMs *n* = 39) with four duplicates; *p < 0.05, 5 (Student's *t*‐test). (K–P) A seahorse assay was performed to investigate the mitochondrial function. Representative traces (K) showing the oxygen consumption rate of 30 wells of PD‐iCMs and Ctrl‐iCMs following the sequential treatments with oligomycin (Oligo, 2 μM), FCCP (1 μM) and rotenone/antimycin A (Rot/Ant, 0.5 μM). Quantification of basal respiration (L), maximal respiration (M), reserve capacity (N), proton leak (O) and ATP production (P). All measurements were normalized to cell counts and presented as mean ± SD with 30 duplicates, *p < 0.05 and ****p < 0.0001, (Student's *t*‐test). (Q,R) Representative images of mitochondrial membrane potential (Q) and ROS level (R) by JC1 and DCFDA, respectively. Three independent repeats were performed per experiment.

Since hypertrophic cardiomyopathy is a particularly common manifestation in PD, we examined whether the derived PD‐iCMs could recapitulate the pathological phenotypes in IOPD. Ctrl‐iCMs and PD‐iCMs were immunostained for cTNT (Figure 3A) and quantitative analysis showed that the average cell surface area was significantly larger in PD‐iCM than Ctrl‐iCM (Figure 3B). TEM showed that sarcomere took a higher percentage of intracellular space in PD‐iCMs than that in Ctrl‐iCMs (Figure 3C,D). Consistent with cardiomyocyte hypertrophy, qPCR showed increased mRNA expression of cardiac hypertrophy markers (ANP, BNP and MYH7) and decreased mRNA expression of MYH6 in PD‐iCMs (Figure 3E–H).

Mitochondrial dysfunction is a common pathogenic mechanism in patients with hypertrophic cardiomyopathy and has been proposed as a disease‐modifying factor of PD.^19^ We hypothesized that mitochondrial impairment underlies the cardiac pathology in PD. Consequently, TEM showed that the average number of mitochondria per visual field, but not the surface area, was markedly decreased in PD‐iCMs than that in Ctrl‐iCMs (Figure 3I,J), suggesting the defect of mitochondrial biogenesis. To investigate the function of mitochondria, the seahorse assay was performed (Figure 3K). The results showed remarkably decreased oxidative phosphorylation in PD‐iCMs relative to that in Ctrl‐iCMs, as shown by the decreased basal, maximal and spare respiration (Figure 3L–N). This dysfunction of mitochondria reduced proton leakage and decreased ATP production in PD‐iCMs (Figure 3O,P). Further analysis showed that mitochondria membrane potential dropped significantly (Figure 3Q), producing an increased ROS level (Figure 3R) in PD‐iCMs compared with that in Ctrl‐iCMs. Overall, lysosome glycogen accumulation impaired the biogenesis and functions of mitochondria, which may contribute to pathogenesis of cardiac pathology in PD.

### 3.5 Impaired mitochondrial fusion and mitophagy partially constituted the underlying mechanism of defective mitochondria in PD‐iCMs

**FIGURE 4:**
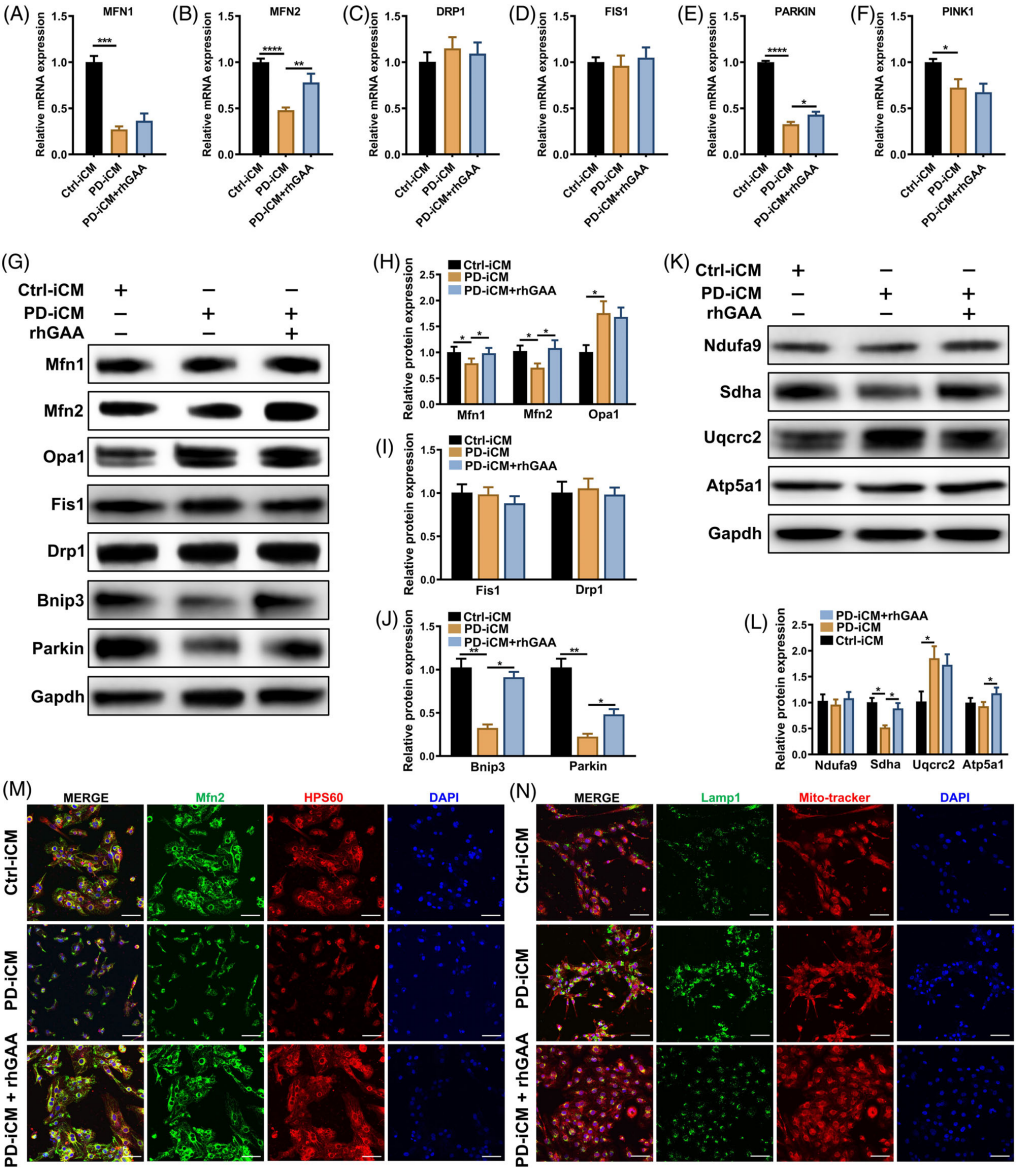
Impaired mitochondrial fusion and mitophagy partially constitute the underlying mechanism of defective mitochondria in PD‐iCMs. RT‐qPCR analysis of the mRNA expression of markers specific for mitochondrial fusion, (MFN1 and MFN2) (A,B), fission, (DRP1 and FIS1) (C,D) and mitophagy (Parkin and PINK1) (E,F). Data are presented as mean ± SD, with three duplicates; ****p < 0.0001, ***p < 0.001 and *p < 0.05 (ANOVA). (G–J) Western blot showing the protein expression of specific markers of mitochondrial fusion, (MFN1, MFN2 and OPA1) (H), fission, (DRP1 and FIS1) (I) and mitophagy (BNIP3 and Parkin) (J). Data are presented as mean ± SD with three independent repeats; **p < 0.01, *p < 0.05 (ANOVA). (K,L) Representative image of western blot for markers of mitochondrial complexes (complex I, NDUFA9; complex II, SDHA; complex III, UQCRC2 and complex V, ATP5A1) (K) and the relative quantification of grayscale values (L). Data are presented as mean ± SD; *p < 0.05 (ANOVA). (M) Representative IF images of mitochondrial markers, MFN2 and HSP60 showing that impaired fusion of mitochondria in PD‐iCMs was alleviated by rhGAA treatment compared with that in Ctrl‐iCMs; scale bar, 50 μm. (N) Representative IF images of lysosomal markers, LAMP1, showing that aggregation of lysosomes in PD‐iCMs, compared with that in Ctrl‐iCMs, was reversed by rhGAA treatment; scale bar, 50 μm. Three independent repeats were performed per experiment.

Mitochondrial dynamics, comprising mitochondrial fusion, fission, biogenesis and mitophagy, determine the mitochondrial morphology, quality, and abundance and considerably affect the development and progression of cardiovascular pathologies. To investigate the detailed mechanism underlying the impaired mitochondria in PD‐iCMs, qPCR was performed to test the mRNA expression of markers for mitochondrial fusion (MFN1 and MFN2), fission (DRP1 and FIS1) and mitophagy (PARKIN, PINK1). It was showed that the impairment of fusion (Figure 4A,B) and mitophagy (Figure 4E,F) in PD‐iCMs which was partially reversed by rhGAA treatment, whereas fission was not apparently changed (Figure 4C,D). These results were supported by the western blot analysis (Figure 4G–J). Decreased expression of the outer membrane fusion markers (Mfn1, Mfn2) but not that of the inner membrane (Opa1) was found in PD‐iCMs compared with Ctrl‐iCMs was partially reversed by rhGAA treatment (Figure 4H) in contrast with fission markers but without significant change (Figure 4I), which was confirmed by the increased interconnected mitochondrial network resulting from rhGAA treatment compare with that in PD‐iCMs as shown by the IF results (Figure 4M). In addition, western blotting showed that impaired mitophagy indicated by the expression of Parkin, Bnip3 in PD‐iCMs was mitigated by rhGAA (Figure 4G,J) which was supported by IF results showing less aggregated lysosome as indicated by Lamp1 expression (Figure 4N). Finally, western blotting evaluation of the protein levels of mitochondrial complexes showed that complex II (Sdha) and V (Atp5a1) were significantly increased in PD‐iCMs treated with rhGAA (Figure 4K,L). These data suggest that impaired mitochondrial fusion, mitophagy and downregulated mitochondrial complexes are partial responsible for the defective mitochondrial function in PD‐iCM, which was partially reversed by the treatment with rhGAA.

### 3.6 rhGAA significantly improved the mitochondrial biogenesis and function and then rescued the hypertrophic phenotype of PD‐iCMs

**FIGURE 5:**
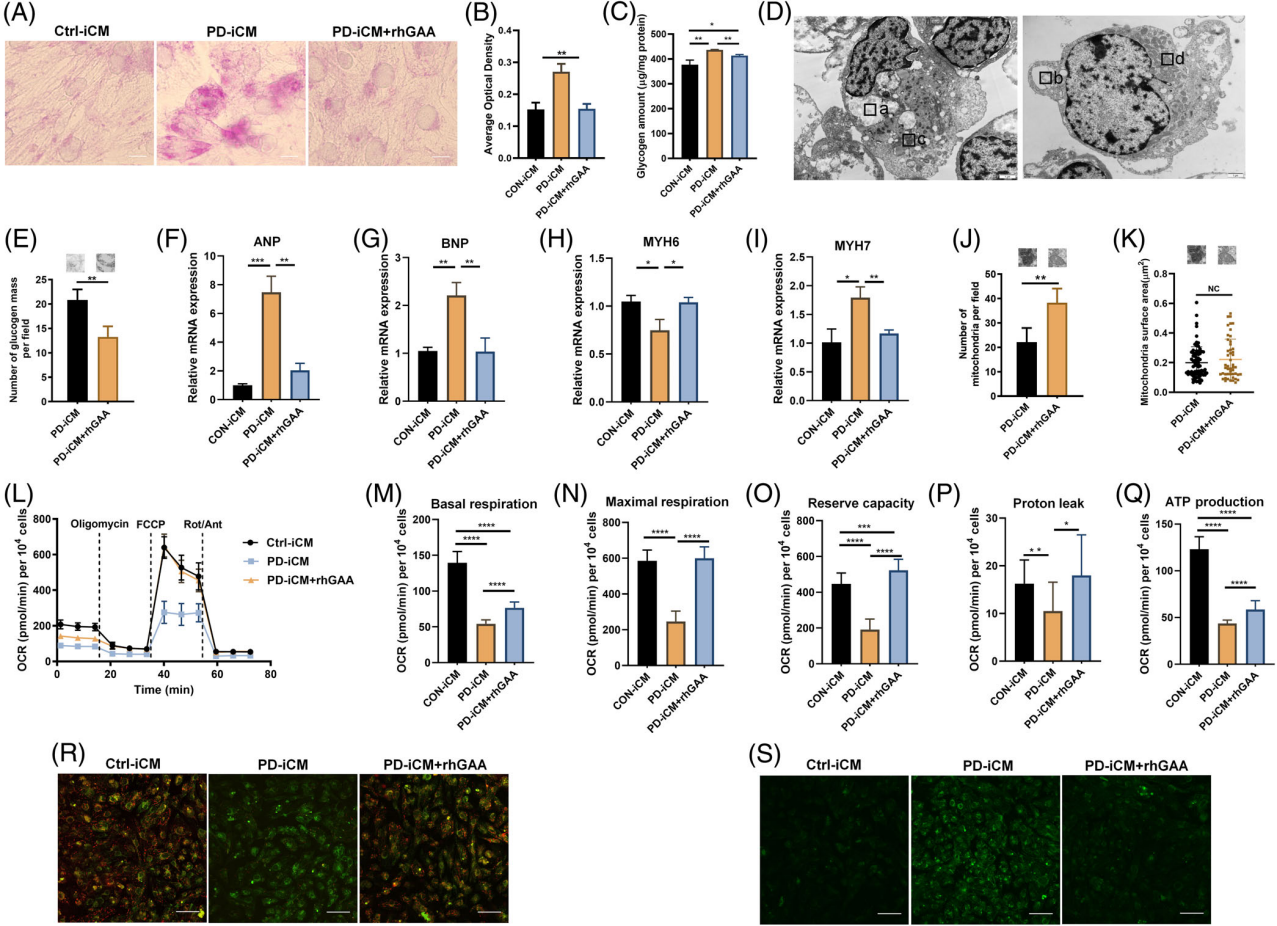
Cardiac hypertrophy was partially reversed with rhGAA treatment in PD‐iCMs. (A,B) Representative images of PAS staining for glycogen from Ctrl‐iCMs and PD‐iCMs with and without rhGAA treatment (A) and quantitation of the average optical density of PAS staining (B). Data are presented as mean ± SD (number of random fields *n* = 5) with three duplicates; **p < 0.01 (ANOVA). (C) Measurement of glycogen content from PD‐iPSCs and Ctrl‐iPSCs. All measurements were normalized to protein concentration and presented as the mean ± SD with three duplicates; *p < 0.05 and **p < 0.01 (ANOVA). (D,E) Representative TEM images showing that glycogen accumulation in PD‐iCMs was significantly mitigated by rhGAA treatment (D). The number of glycogen mass was quantitated using ImageJ (E). Data are presented as the mean ± SD (cell number: Ctrl‐iCMs *n* = 19, PD‐iCMs *n* = 16) with three duplicates; **p < 0.01 (ANOVA). (F–I) RT‐qPCR analysis of the mRNA expression of markers of cardiac hypertrophy, ANP (F), BNP (G), MYH6 (H) and MYH7 (I) from PD‐iCMs and rhGAA‐treated PD‐iCMs. Data are presented as mean ± SD with three duplicates; *p < 0.05, **p < 0.01 and NS, not significant (Student's *t*‐test). (J,K) TEM images showing the number (J) and area (K) of mitochondria from Ctrl‐iCMs and PD‐iCMs without and with rhGAA treatment. Data are presented as the mean ± SD (cell number: Ctrl‐iCMs *n* = 75, PD‐iCMs *n* = 39) with four duplicates; *p < 0.01 (ANOVA). (L–Q) A seahorse assay was performed to investigate the mitochondrial function. Representative traces (L) showing the oxygen consumption rate of 30 wells of Ctrl‐iCMs and PD‐iCMs with and without rhGAA treatment following the sequential treatments with oligomycin (Oligo, 2 μM), FCCP (1 μM) and rotenone/antimycin A (Rot/Ant, 0.5 μM). Quantification of basal respiration (M), maximal respiration (N), reserve capacity (O), proton leak (P) and ATP production (Q). All measurements were normalized to cell counts and presented as mean ± SD; *p < 0.05 and ****p < 0.0001, (Student's *t*‐test). (R,S) Representative images of mitochondrial membrane potential (R) and ROS level (S) by JC1 and DCFDA respectively; scale bar, 50 μm. Three independent repeats were performed per experiment.

rhGAA is the first‐line and only effective treatment for patients with PD. Since PD‐iPSCs recapitulated the cardiac hypertrophy phenotype of patients with PD, we, therefore, examined whether the application of rhGAA could rescue the cardiomyocyte hypertrophy phenotype in PD‐CMs. The results showed that the accumulation of lysosomal glycogen was markedly reduced in PD‐iCMs after a 6‐day treatment as shown by the PAS staining (Figure 5A,B), quantitative detection of glycogen (Figure 5C) and TEM (Figure 5D,E). In agreement, the increased mRNA expression of cardiac hypertrophic marker genes ANP and BNP, but not MYH6 or MYH7, were significantly ameliorated (Figure 5F–I). Moreover, TEM results showed that the number of mitochondria but not the surface area, was markedly increased in PD‐iCMs + rhGAA group than that in PD‐iCMs (Figure 5J,K). The seahorse assay (Figure 5L) showed that the oxidative phosphorylation was markedly decreased in PD‐iCMs relative to that in Ctrl‐iCMs which was partially rescued by the rhGAA treatment as evidenced by the increased basal, maximal, and spare respiration and subsequent increase in proton leakage and ATP production (Figure 5M–Q). Further analysis showed the depolarized mitochondria membrane potential (Figure 5R) and subsequently increased ROS levels (Figure 5S) in PD‐iCMs compared with that in Ctrl‐iCMs were also mitigated by rhGAA treatment. Overall, these results suggest that in addition to the alleviation of lysosome glycogen accumulation, rhGAA could partially reverse the defective biogenesis and functions of mitochondria, which may contribute to pathogenesis of cardiac pathology in PD.

## 4 DISCUSSION

PD is a rare autosomal recessive genetic disease caused by mutations in the *GAA* gene, producing a deficiency in GAA activity. Clinically, PD can be mainly classified mainly into IOPD and LOPD based on the time of onset and residual GAA enzyme activity.^20^, ^21^ We generated two iPSC clones from one family, one from the proband with IOPD carrying R608X and E888X compound mutations in the GAA gene,^16^ and the other from the mother carrying an E888X mutation without a disease phenotype.^17^ Both iPSC clones possess similar characteristics to those of pluripotent stem cells. The PD‐iPSCs showed decreased expression of GAA at both the mRNA and protein levels, and an extremely low GAA enzyme activity. Furthermore, the PD‐iPSCs showed strong PAS‐positive granular structures in their cytoplasm, which is a typical pathological feature of PD. Our results are consistent with previous reports where the typical pathological features manifested at the early stage during the development of IOPD in both mouse and human PD‐iPSCs.^20^, ^22^, ^23^

Patients with IOPD usually manifest obvious cardiovascular complications, such as cardiac hypertrophy and heart failure.^2^ Owing to the difficulty in procuring cardiac tissues, the research in the development of a treatment for IOPD has remained still limited. Although GAA‐knockout mice and cardiomyocytes derived from iPSCs have been used to study the pathophysiological characteristics of PD,^23^, ^24^ the phenotypic features in the development process are not fully characterized. After successfully reprograming, both Ctrl‐iPSCs and PD‐iPSCs were differentiated into robust beating cardiomyocytes. Characterization of these induced cardiomyocytes showed that both sets possessed similar phenotypical features of cardiomyocytes, and expressed specific cardiomyocyte markers ACTIN2 and TNNT2. Furthermore, the PD‐iCMs faithfully recapitulated several PD‐specific cardiac pathophysiological characteristics including coarse paranuclear Lamp1 positive granules, representing the enlargement of the lysosomal compartment; and coarse cytosolic GAA‐positive granules. Compared with Ctrl‐iCMs, PD‐iCMs showed significantly decreased GAA expression and enzyme activity, and increased PAS‐positive lysosome glycogen accumulation. Using IOPD‐iPSCs‐derived cardiomyocytes, Raval et al. indicate that deficiency of Golgi‐based glycosylation of LAMP may partially reveal the mechanism of lysosome dysfunction.^25^ Further investigation is necessary to assess the correlation between lysosomal signalling pathways and GAA enzyme function in PD‐iCMs.

Enzyme replacement therapy (ERT) with glucosidase alfa (Myozyme) was initiated in 2006, and was found to improve outcomes in patients with classic infantile PD. Based on the IODP‐iPSC derived cardiomyocyte model, we found that the phenotype of cardiac hypertrophy and dysfunction of mitochondria could be significantly alleviated using rhGAA. In addition to the ERT strategy, gene therapy is another promising strategy. Sato et al. showed that the delivery of lentiviral *GAA* improved GAA enzyme activity and decreased glycogen accumulation in iPSCs. Furthermore, the efficacy of gene therapy could be maintained following the cardiomyocyte differentiation, suggesting the potential of gene therapy in the treatment of PD in the future.^26^

Cardiac hypertrophy secondary to PD is classified as hypertrophic cardiomyopathy because of the marked thickening of the ventricular walls and associated hyperdynamic systolic function with outflow tract obstruction. Since perturbed metabolic signalling and mitochondrial dysfunction are common pathogenic mechanisms in patients with hypertrophic cardiomyopathy,^7^ it is reasonable to speculate that mitochondria may contribute significantly to the development of cardiac hypertrophy in patients with PD. Indeed, Lim et al. demonstrated mitochondrial dysfunction was associated with aberrant energy metabolism in skeletal muscle biopsy of patients with PD and animal models.^10^ Huang et al. observed the presence of obviously swollen cristae and mitochondrial dysfunction including decreased glycolysis and XPHOS in iPSC‐derived from the fibroblasts of patients with PD.^20^ Moreover, accumulating evidence has shown the abnormal morphology of mitochondria, such as deformed, abnormally large and aggregated mitochondria, and the presence of dense granular materials and inclusion bodies in intercristae space in skeletal muscle biopsies from PD patients with PD and PD mice.^27^ However, whether mitochondrial dysfunction underlies the development of cardiac hypertrophy in patient with PD in unknown. Herein, we expanded these early discoveries that deformed mitochondria were often observed in muscle biopsy of patients with PD to show decreases in the number of mitochondria, consumption of oxygen, and production of ATP and a collapse in membrane potential of mitochondria with increased levels of ROS in PD‐iCMs compared with that in Ctrl‐iCMs.^10^ Our results lend support to the hypothesis that mitochondria dysfunction significantly contributes to the pathogenesis of hypertrophic cardiomyopathy in PD and mitochondria could be a potential therapeutic target. Nevertheless, the causal relationship between mitochondrial impairment and cardiac hypertrophy in PD remains to be tested.

Although studies reported that impaired mitochondria have been observed in PD‐derived iPSCs and skeletal muscle biopsies of patients with PD and PD‐modelled mice, the underlying mechanism has yet to be elucidated. Our data showed that at least the defective mitochondrial fusion and mitophagy partially mediated the impairment of mitochondria in cardiac muscles from patients with PD. The mitochondrial morphology, quality and abundance are recognized as being determined by the mitochondrial dynamics comprising mitochondrial fusion, fission, biogenesis and mitophagy.^28^ Imbalanced or damaged mitochondrial dynamics have been implicated in the development and progression of cardiovascular pathologies.^24^, ^29^ Mitochondrial fusion connects neighbouring depolarized mitochondria and mixes their contents to maintain membrane potential. Conversely, mitochondrial fission segregates damaged mitochondria from intact ones with the damaged part of mitochondria undergoing mitophagy while the intact part undergoes fusion. Mitochondria fusion is considered to be beneficial for the heart, especially under stress conditions because this consolidates the mitochondria's supply of energy. Given that lysosomes play a crucial role in most of the processes that allow removal of dysfunctional mitochondria (mitophagy),^30^, ^31^ it is possible that over‐accumulation of glycogen induces dysfunctional lysosomes that can no longer clear damaged mitochondria via mitophagy and fusion, leading to defective mitochondria in PD‐iCMs. Therefore, it is vital to explore the mechanisms linking lysosomal and mitochondrial dysfunction that may illuminate the development of novel therapeutic strategies for PD.

In conclusion, we used iPSC‐iCMs derived from a patient with IOPD to find impaired mitochondrial functions including reduced mitochondrial biogenesis, oxygen consumption and ATP production were involved in the cardiac pathology in IOPD‐iCMs. Mechanistic analysis indicated that impaired fusion and autophagy of mitochondria and declined expression of mitochondrial complexes underlies the mechanism of dysfunctional mitochondria. This was alleviated by supplementation with rhGAA, leading to improved mitochondrial function and concomitantly mitigated cardiac pathology. Our discoveries support the newly proposed theory that mitochondria dysfunction is associated with hypertrophic cardiomyopathy of IOPD. Targeting the mitochondrial may therefore serve as a novel candidate adjuvant therapeutic strategy in future PD clinical practice.

## AUTHOR CONTRIBUTIONS

**Wenjun Huang**: Conception and design; literature research; experimental studies; collection and/or assembly of data; data analysis; interpretation and manuscript writing; **Rui Zhou**: Molecular biology experiments and manuscript revision/review; **Congshan Jiang**: Literature research and data analysis/interpretation; **Jie Wang**: Statistical analysis; **Yafei Zhou**: Cellular and function experiment; **Xiaoyan Xu**: Biochemical experiment; **Tao Wang**: Data analysis; **Anmao Li**: Manuscript revision/review; **Yanmin Zhang**: Conception and design; interpretation; manuscript writing and editing and revision/review and final approval of manuscript. All authors have read and approved the final manuscript.

## CONFLICT OF INTEREST STATEMENT

The authors declare no conflicts of interest.

## Supporting information


**Figure S1.** Characterization of IOPD patients. (A) Genetic pedigree of the family. II‐1 is the proband carrying compound heterozygous mutations. (B) Sanger sequencing shows the proband's compound mutations of c.1822C>T, p.R608X and c.2662G>T, p.E888X in the GAA gene. (C,D) Cardiac hypertrophy of the proband by echocardiography (C) and electrocardiography (D).

**Figure S2.** Characterization of the control iPSC from the mother (Ctrl‐iPSC). (A) Representative image showing the typical morphology of Ctrl‐iPSC. Scale bar, 50 μm. (B) Sanger sequencing of the GAA gene containing heterozygous mutation of c.2662G>T, p.E888X in Ctrl‐iPSC. (C) Karyotype analysis showing Ctrl‐iPSC has normal karyotypes. (D–E) Pluripotent markers of iPSC, NANOG, OCT4, SOX2 and TRA‐1‐60 (D), and self‐renewal marker, Ki67 (E) by IF assay. Scale bar, 50 μm. (F) The differentiation of Ctrl‐iPSC into three germ layers iPSC(ectoderm, PAX6; endoderm, FOXA2; mesoderm, SOX17) in vitro trilineage differentiation assay. Scale bar, 50 μm.

**Figure S3.** Identification of the iPSC derived cardiomyocytes. (A) IF showing the expression of cardiomyocytes marker cTNT in Ctrl‐iCM and PD‐iCM. Scale bars, 20 μm. (B) Quantitative RT‐PCR analysis for cardiomyocytes markers (ACTN2 and TNNT2) from Ctrl‐iCM and PD‐iCM. (C‐D) Representative images of flow cytometry (C) and quantitative data (D) from Ctrl‐iCM and PD‐iCM. (E–G) Quantitative RT‐PCR analysis for cardiomyocytes maturity markers, sarcomeric structure (E), calcium handling (F) and ion channels (G). Data are presented as ‘mean ± SD’. *p < 0.05 and **p < 0.01. (Student's *t*‐test, *n* = 3).

**Table S1.** Primer sets for qRT‐PCR analysis.

**Data S1:** Supporting Information.

## Data Availability

Datasets used and/or analysed during the current study are available from the corresponding author on reasonable request.
