# Supplementary figures and images for: Mitochondrial dysfunction is associated with hypertrophic cardiomyopathy in Pompe disease‐specific induced pluripotent stem cell‐derived cardiomyocytes
Source: Cell Prolif. 2023 Nov 2;57(4):e13573. doi: 10.1111/cpr.13573 (PMC10984102; doi:10.1111/cpr.13573)

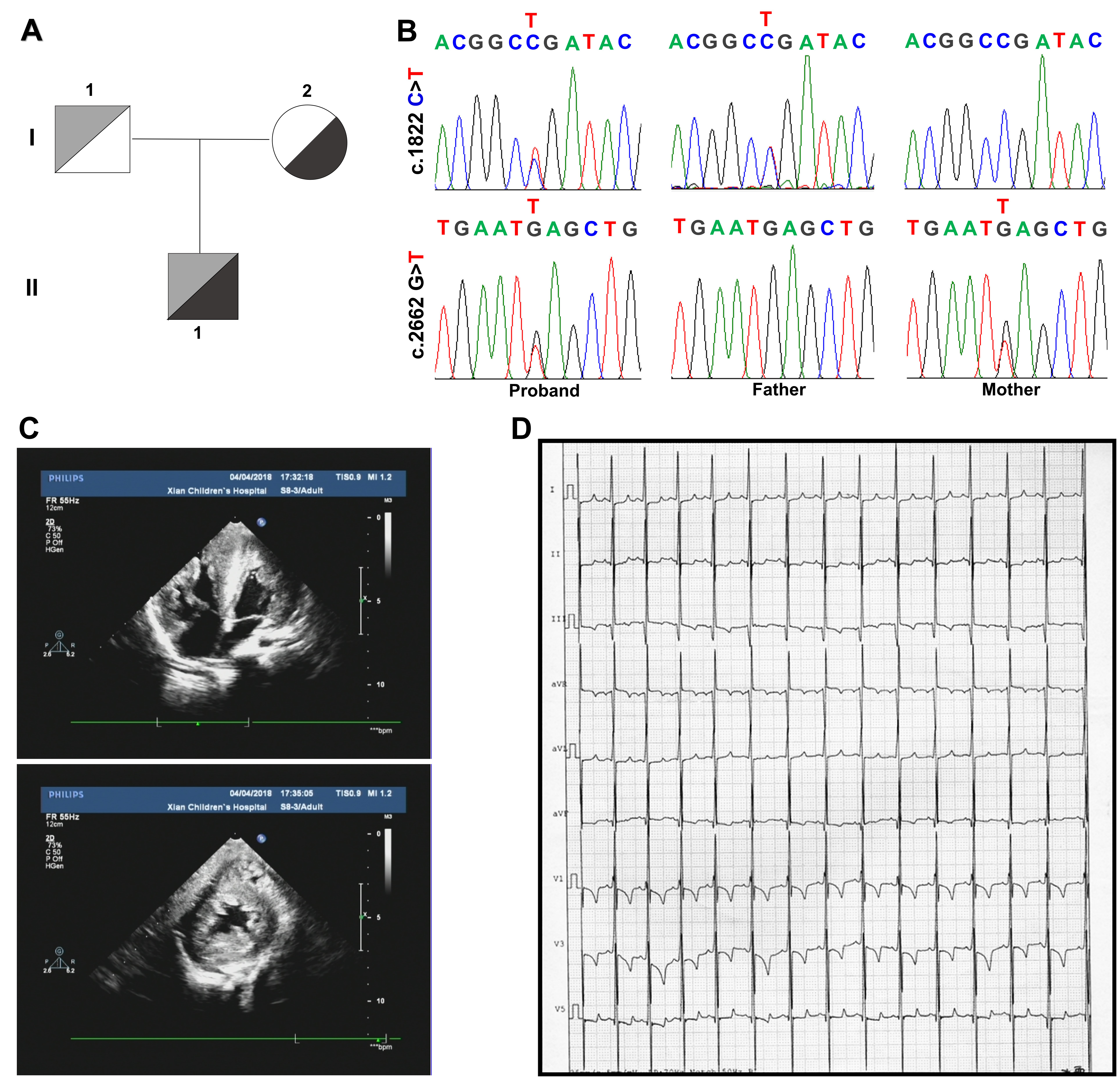

Supplement: Supplementary file 1 — Figure S1. Characterization of IOPD patients. (A) Genetic pedigree of the family. II‐1 is the proband carrying compound heterozygous mutations. (B) Sanger sequencing shows the proband's compound mutations of c.1822C>T, p.R608X and c.2662G>T, p.E888X in the GAA gene. (C,D) Cardiac hypertrophy of the proband by echocardiography (C) and electrocardiography (D). [file CPR-57-e13573-s002.jpg]

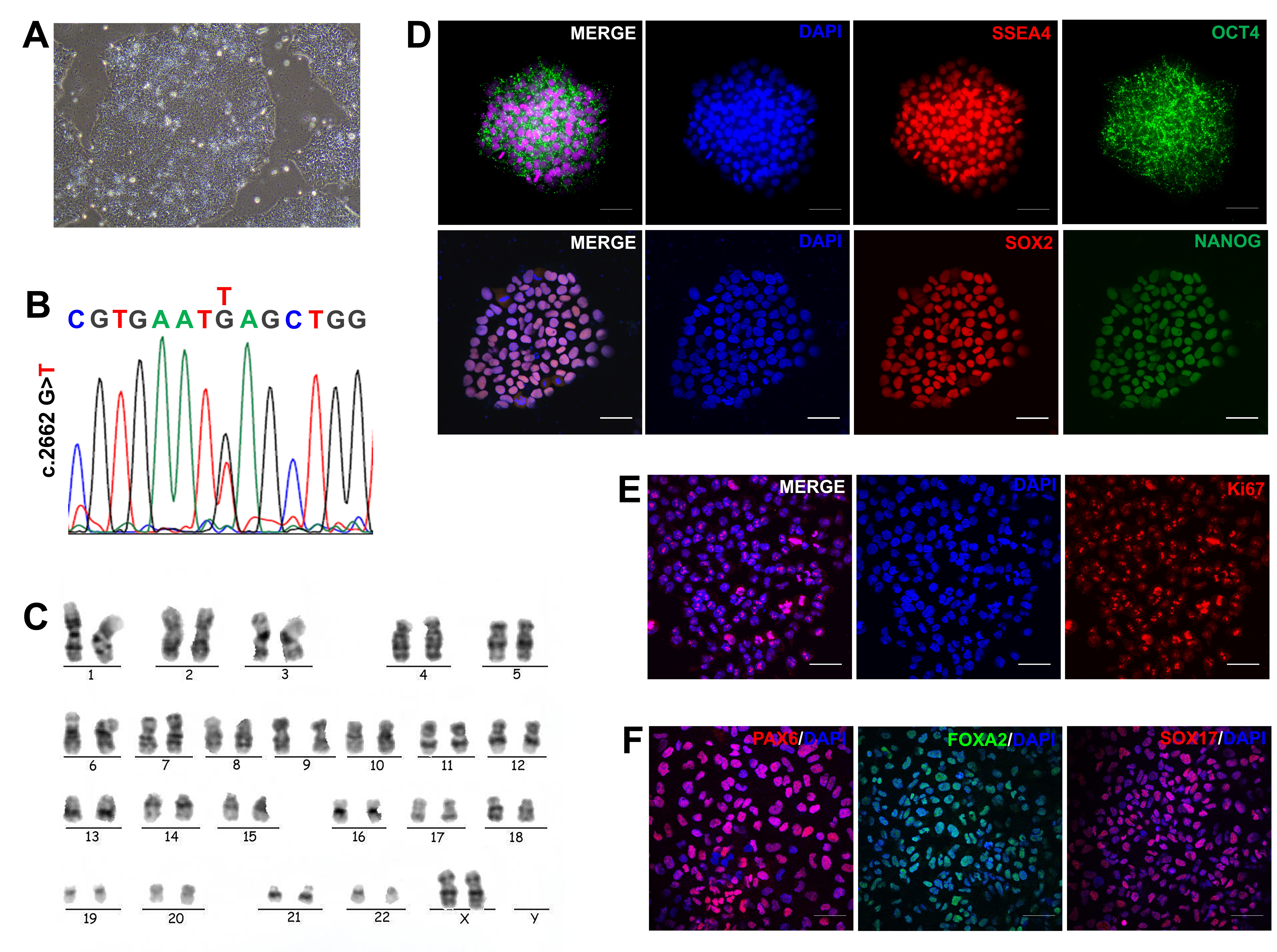

Supplement: Supplementary file 2 — Figure S2. Characterization of the control iPSC from the mother (Ctrl‐iPSC). (A) Representative image showing the typical morphology of Ctrl‐iPSC. Scale bar, 50 μm. (B) Sanger sequencing of the GAA gene containing heterozygous mutation of c.2662G>T, p.E888X in Ctrl‐iPSC. (C) Karyotype analysis showing Ctrl‐iPSC has normal karyotypes. (D–E) Pluripotent markers of iPSC, NANOG, OCT4, SOX2 and TRA‐1‐60 (D), and self‐renewal marker, Ki67 (E) by IF assay. Scale bar, 50 μm. (F) The differentiation of Ctrl‐iPSC into three germ layers iPSC(ectoderm, PAX6; endoderm, FOXA2; mesoderm, SOX17) in vitro trilineage differentiation assay. Scale bar, 50 μm. [file CPR-57-e13573-s004.jpg]

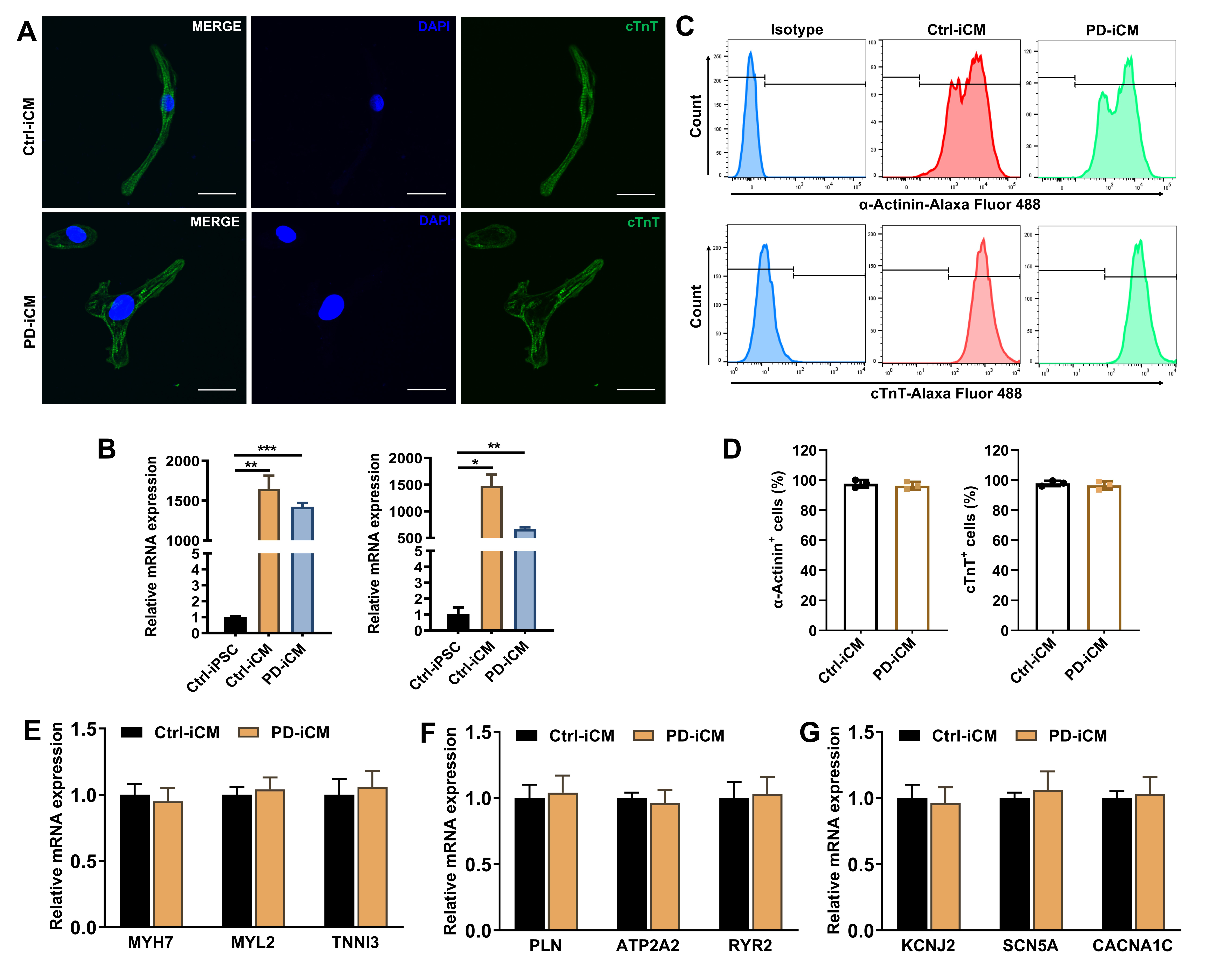

Supplement: Supplementary file 3 — Figure S3. Identification of the iPSC derived cardiomyocytes. (A) IF showing the expression of cardiomyocytes marker cTNT in Ctrl‐iCM and PD‐iCM. Scale bars, 20 μm. (B) Quantitative RT‐PCR analysis for cardiomyocytes markers (ACTN2 and TNNT2) from Ctrl‐iCM and PD‐iCM. (C‐D) Representative images of flow cytometry (C) and quantitative data (D) from Ctrl‐iCM and PD‐iCM. (E–G) Quantitative RT‐PCR analysis for cardiomyocytes maturity markers, sarcomeric structure (E), calcium handling (F) and ion channels (G). Data are presented as ‘mean ± SD’. *p < 0.05 and **p < 0.01. (Student's t‐test, n = 3). [file CPR-57-e13573-s003.jpg]
