# Supplementary material for: Mitochondrial dysfunction is associated with hypertrophic cardiomyopathy in Pompe disease‐specific induced pluripotent stem cell‐derived cardiomyocytes
Source: Cell Prolif. 2023 Nov 2;57(4):e13573. doi: 10.1111/cpr.13573 (PMC10984102; doi:10.1111/cpr.13573)
Supplement: Supplementary file 4 — Table S1. Primer sets for qRT‐PCR analysis. [file CPR-57-e13573-s001.docx]

Table1. Primer sets for qRT-PCR analysis.

| Gene | Direction | Sequence (5’-3’) | Product size (bp) |
| --- | --- | --- | --- |
| TNNT2 | Forward | TTCACCAAAGATCTGCTCCTCGCT | 91 |
|  | Reverse | TTATTACTGGTGTGGAGTGGGTGTGG |  |
| ACTN2 | Forward | ATGGCCTTGGACTCTGTGC | 167 |
|  | Reverse | GGTGTTCACGATGTCTTCAGC |  |
| ANP | Forward | GCCTAGGGACAGACTGCAAG | 170 |
|  | Reverse | GGCGAGGAAGTCACCATCAA |  |
| BNP | Forward | TGGAAACGTCCGGGTTACAG | 122 |
|  | Reverse | CTTCCAGACACCTGTGGGAC |  |
| MYH6 | Forward | CTCCGTGAAGGGATAACCAGG | 91 |
|  | Reverse | CTGACTTGCGGAGGTACTGG |  |
| MYH7 | Forward | AGACACACTTGAGTAGCCCA | 153 |
|  | Reverse | CATCAGGCACGAAGACATCCT |  |
| GAPDH | Forward | TCGGAGTCAACGGATTTGGT | 181 |
|  | Reverse | TTCCCGTTCTCAGCCTTGAC |  |
| MFN1 | Forward | GCTGTTGCCGGGTGATAGTT | 89 |
|  | Reverse | GCCTTCTTAGCCAGCACAAAG |  |
| MFN2 | Forward | AAGGTGAAGCGCAATGTCC | 88 |
|  | Reverse | ATTCACCTCAGCCATGTGTCTC |  |
| DRP1 | Forward | TGCTTCCCAGAGGTACTGGA | 180 |
|  | Reverse | TCTGCTTCCACCCCATTTTCT |  |
| FIS1 | Forward | TCTGCTCCCCTGAGATTCGT | 74 |
|  | Reverse | GCCACAGCCCCGTTTTATTT |  |
| PARKIN | Forward | AACTCCAGCCATGGTTTCCC | 119 |
|  | Reverse | CCTGCGAAAATCACACGCAA |  |
| PINK1 | Forward | TTGCCCCTAACACGAGGAAC | 95 |
|  | Reverse | ACGTGCTGACCCATGTTGAT |  |
| MYL2 | Forward | ATTCTTCTCGGGAGGCAGTG | 166 |
|  | Reverse | TGAAGCCATCCCTGTTCTGG |  |
| TNNI3 | Forward | CCTCCAACTACCGCGCTTAT | 77 |
|  | Reverse | CTGCAATTTTCTCGAGGCGG |  |
| PLN | Forward | GCCTCAACAAGCACGTCAAA | 171 |
|  | Reverse | TCCTGTCTGCATGGGATGAC |  |
| RYR2 | Forward | AAGATCATGCAGCCAGAGCC | 161 |
|  | Reverse | ACAACCAGGACGACTCCAAC |  |
| KCNJ2 | Forward | CCTGGCTTTCGTCCTGTCAT | 122 |
|  | Reverse | TGAAGCTGTTGACCTCGGAC |  |
| SCN5A | Forward | CCAGCAAGCAGGAATCCCA | 194 |
|  | Reverse | CTCGGAGCAACTGTCCTCTG |  |
| CACNA1C | Forward | GCTTATGGGGCTTTCTTGCAC | 108 |
|  | Reverse | ACTGGACTGGATGCCAAAGG |  |
