# Supplementary material for: Mitochondrial dysfunction is associated with hypertrophic cardiomyopathy in Pompe disease‐specific induced pluripotent stem cell‐derived cardiomyocytes
Source: Cell Prolif. 2023 Nov 2;57(4):e13573. doi: 10.1111/cpr.13573 (PMC10984102; doi:10.1111/cpr.13573)
Supplement: Supplementary file 5 — Data S1: Supporting Information. [file CPR-57-e13573-s005.docx]

**Experimental procedures**

**Reagents**

The source information of reagents and cell lines used in the study are as follows: Patient-specific iPSC (PD-iPSC) and healthy control iPSC (Ctrl-iPSC) were generated through introduction of the four reprogramming factors, Klf-4, Oct 3/4, Sox-2, and c-Myc using Sendai virus. Cell culture media RPMI/1640 (11875093) and DMEM/F12 (11320082) were from Thermo Fisher Scientific, iPSC BioCISO (BC-PM0001) from OSINGLAY BIO, China, and Aaccutase (7920) from Gibco. GSK3 inhibitor CHIR99021 (S1263) was from Sigma, Wnt inhibitor IWP2 (3533) and ROCK inhibitor Y27632 (1254) from Tocris Bioscience, recombinant GAA, Myozyme from Genzyme, B27 supplement with (17504-044) or without insulin from Thermo Fisher Scientific, Matrigel (354277) from Corning, TRIzol Reagent (15596026) from Thermo Fisher Scientific, and Real-time PCR reagents (208056) from Qiagen. All primers/oligos were synthesized by Sangon Biotech, China, and listed in Supplemental Table 1. All other reagents, unless specified otherwise, were products of Sigma.

**iPSC reprogramming and culture**

Human peripheral blood mononuclear cells (PBMC) were harvested from the PD patient’s mother and father. Reprogramming to iPSCs from PBMC was performed using the commercial Sendai virus reprogramming kit (A34546, Invitrogen, USA) containing the four Yamanaka factors OCT4, SOX2, KLF4 and CMYC. iPSC colonies emerged as early as 7 days after virus transduction, which were then picked, expanded and characterized. The iPSC lines were cultured with the iPSC medium, BioCISO in plates pre-coated with Matrigel (1:50). When reaching about 80% confluence, the iPSCs were trypsinized and subcultured in a ratio between 1: 4 and 1: 8 in the same medium containing 5μM of Y27632.

**Karyotype analysis**

iPSC cells during the exponential growth phase were treated with colchicine (0.2μg/ml) for 2h and then harvested. After treatment with a hypotonic KCl solution, the cells were fixed with acetic acid/methanol solution and then dropped onto slides for chromosome analysis using the Trypsin-Giemsa banding technique[[12](#_ENREF_12)].

**Differentiation of three germ layers *in vitro***

The iPSC cells at the logarithmic phase were seeded into 12-well plate containing coverslips pre-coated with Matrigel. Three germ layers were differentiated using the STEMdiff™ Trilineage Differentiation Kit (Stem Cell Technologies) following manufacturer’s protocol. At day 5 or 7, immunostaining was performed to evaluate the expression of the markers of all three germ layers.

**Cardiomyocytes differentiation from iPSC**

Cardiomyocytes differentiation was performed using the small molecule-based differentiation protocol as previously reported[[13](#_ENREF_13)]. Briefly, 80–90% confluent iPSCs were harvested using Accutase and resuspended with iPSC-maintaining medium at a density of 0.5 × 10^5^ cells per milliliter (mL). Two mL of the cell suspension was seeded per well in a 12-well plate pre-coated with Matrigel, which was arbitrarily defined as minus day 4. Starting from day 0, the medium was refreshed, sequentially, with insulin-free RPMI/B27 containing 10 μM of CHIR99021 (GSK3 inhibitor) for 24 h (day 0), containing no GSK3 inhibitor for 48 h (day 1-2), containing 5 μM of IWP2 (Wnt inhibitor) for 48 h (day 3-4), and containing no Wnt inhibitor for another 48 h (day 5 to 6). From day 7, the medium was refreshed to RPMI/B27 containing insulin every 3 days. The beating cardiomyocytes can emerge as early as from day 8. The induced cardiomyocytes were purified using the metabolic-selection method as previously described[[14](#_ENREF_14)].

**RNA isolation and qPCR**

Total RNA was isolated using TRizol method (15596026, Invitrogen, USA). Five hundred (500) nanograms of total RNA was reversely transcribed with the PrimeScript™ RT Master Mix (RR036, Takara, Japan) following the manufacturer’s instructions. The cDNA was used for real-time PCR using TB Green™ Fast qPCR Mix (RR430, Takara, Japan). The PCR conditions were 95 °C for 2 min, followed by 40 cycles of 95 °C for 20″ and 60 °C for 15″. All primers are listed in Suppl. Table 1. The expression of target gene was normalized to that of GAPDH and calculated using the 2^-ΔΔCt^ method.

**Immunofluorescence**

The iPSC cells and their derived cardiomyocytes were seeded in cell culture plate containing coverslips pre-coated with Matrigel for 48 h. Cells were fixed with 4% (w/v) paraformaldehyde (PFA) for 15 min, and permeabilized/blocked with DPBS containing 0.5% Triton X-100 (A600198-0500, BBI, China) and 5% BSA (A600332-0025, BBI, China) for 30 min at room temperature. The cells were then incubated with the following primary antibodies: anti-OCT4 antibody (#2750, CST, USA), anti- NANOG antibody (#3580, CST, USA), anti-SSEA4 antibody (#4755, CST, USA), anti-SOX2 antibody (ab55740, abcam, USA), anti-TRA-1-60 antibody (#4746, Cell Signaling Technology, USA), anti-Ki67 antibody (ab15580,abcam, USA), anti-cTNT antibody from mouse (MS-295-P1, Thermo Fisher Scientific, USA), anti-Lamp1 antibody from rabbit (#9091, CST, USA), anti-Alpha-glucosidase antibody (ab15580, abcam, USA), anti-cTNT antibody from rabbit (15513-1-AP, Proteintech, China), anti-HSP60 antibody (MA3-012, Thermo Fisher Scientific, USA), anti-Mfn2 antibody (#9482, CST, USA), for 1 h at RT, followed by incubation with the following species-specific fluorescence-conjugated secondary antibodies: Alexa fluor 488 labeled goat anti-mouse IgG (A-11001, Invitrogen, USA), Alexa fluor 488 labeled goat anti-rabbit IgG (A-11008, Invitrogen, USA), Alexa fluor 594 labeled goat anti-mouse IgG (A-11005, Invitrogen, USA), and Alexa fluor 594 labeled goat anti-rabbit IgG (R37177, Invitrogen, USA) for 1 h at RT. The cells were washed with PBS, and nuclei were counterstained using DAPI (4083, CST, USA) for 15 min at RT. After rinsing with PBS, the coverslips were mounted and visualized under confocal microscope (OLYMPUS FLUOVIEW, FV3000, Japan).

**Flow cytometry**

Cardiomyocytes were harvested by digestion with 0.25% trypsin into single cell suspension and washed with PBS. Cells were fixed with 4% formaldehyde for 15 min at room temperature. Permeabilization and blocking was performed in permeabilization buffer (0.3% Trito X-100 in PBS with 0.5% BSA) for 30 min. Cells were then incubated with anti-cTnT antibody (anti-cTnT antibody from mouse (MS-295-P1, Thermo Fisher Scientific, USA), anti-α-Actinin antibody from mouse (A7811, Sigma, USA), then washed with PBS, and followed by incubation with the species-specific fluorence-conjugated secondary antibodies, alexa fluor 488 labeled goat anti-mouse IgG (A-11029, Invitrogen, USA) for 30 min at room temperature. Cells were analyzed using flow cytometry machine (651155, BD FACS Verse, BD Bioscience, USA) according to the manufacturer's protocol.

**Western blot assay**

Cells were lysed using SDS lysis buffer supplemented with proteinase inhibitors, sonicated and boiled for 10 min. Lysates were spun at top speed for 1 min at room temperature and supernatants were quantitated. Twenty μg of lysate was resolved with 10% SDS-PAGE, and transferred onto PVDF membrane (Millipore, USA). The membrane was blocked with 5% milk in TBST for 1 hour, probed with first antibody:

anti-Mfn1 antibody (#14739, CST, USA), anti-Mfn2 antibody (#9482, CST, USA),

anti-Opa1 antibody (#80471, CST, USA), anti-Fis antibody (10956-1-AP, Proteintech, China), anti-Drp1 antibody (#8570, CST, USA), anti-Bnip3 antibody (#44060, CST, USA), anti-Parkin antibody from mouse (#4211, CST, USA), anti-ndufa9 antibody (29621-1-AP, Proteintech, China), anti-Sdha antibody (14865-1-AP, Proteintech, China), anti-Uqcrc2 antibody (14742-1-AP, Proteintech, China), anti-Atp5a1 antibody (14676-1-AP, Proteintech, China), anti-Gapdh antibody from rabbit (#5174, CST, USA) for overnight at 4°C, washed, and followed by horseradish peroxidase-conjugated secondary antibody incubation for 1 hour. The membrane was then developed with Superglow ECL, image was acquired, and relative expression normalized to that of GAPDH was analyzed by densitometry using the Image J software.

**GAA activity assay**

Cells were harvested and resuspended into sterile water. After 5 cycles of freeze-thaw, the supernatant was collected, and protein concentration was determined by BCA protein assay kit. GAA enzyme activity was measured by Alpha-Glucosidase Activity Assay Kit (ab174093, Abcam) following the vendor’s instruction.

**Periodic Acid-Schiff (PAS) Staining**

PAS staining was performed as previously described[[15](#_ENREF_15)]. Briefly, cells grown on coverslips were fixed with 5% glacial acetic acid in 96% EtOH, rinsed with tap water for 1 min, and immersed in periodic acid solution for 5 min. The coverslips were then rinsed with distilled water 3 times and immersed in Schiff’s reagent for 10 minutes at room temperature. After washing with slowly running tap water for 5 minutes, cells were counterstained in hematoxylin solution for 90 seconds, and rinsed with tap water for another 5 minutes. The coverslips were mounted with fluorescent mounting medium (DAKO), completely dried and observed with light microscope (BX43, OLYMPUS, Japan) in bright field. Twelve non-overlapping microscopic fields from each group were selected, and the average optical density of staining area was calculated using ImageJ software.

**Measurement of cells glycogen contents**

Commercial glycogen assay kit (ab65620, Abcam, USA) was used to measure the glycogen levels in iPSC and iCM according to the instruction manual. Briefly, homogenized cell samples mixed with 2μl of Hydrolysis Enzyme Mix in the 96-well plate was incubated for 30min at room temperature. Following adding 50μl of Reaction Mix for each reaction, well-mixed samples were incubated at room temperature for 30min protected room light. O.D value at 570 nm was measured immediately on a microplate reader. Concentration of Glycogen in the test samples is calculated and normalized with total protein concentrations.

**Transmission electron microscopy**

Cells were pelleted by centrifugation, which were resuspended with 3% glutaraldehyde in DPBS and incubated for 5 minutes at 4℃. The cells were fixed with 2% osmium tetroxide, rapidly dehydrated with graded acetones, and embedded in epoxy resin. Subsequently, the ultrathin sections of the embedded blocks were stained with uranyl acetate and lead citrate. The samples were examined under transmission electron microscope (JEM-1400FLASH, Japan) and images were analyzed using imageJ software.

**Seahorse**

Equal number of isolated cardiomyocytes were seeded in microplates (Seahorse Bioscience, Billerica, MA), which were then treated sequentially with 1 μM antimycin A, 1 μM oligomycin, and 0.5 μM FCCP at RT, the oxygen consumption rate (OCR) was detected using the extracellular flux analyzer (Seahorse Bioscience, Billerica, MA). Basal respiration represents the baseline value of oxygen consumption before, whereas the maximal respiration represents the maximum OCR value after, the treatment with FCCP. Spare respiratory capacity was calculated by recording the OCR response to FCCP, which was then divided by the basal respiration. The OCR was normalized to the cellular protein concentration.

**Reactive Oxygen Species (ROS) Analysis**

Cellular ROS was measured using the ROS assay kit (Abcam, Cambridge, MA, USA). Briefly, 2′,7′-dichlorofluorescein diacetate (DCFDA), a fluorogenic dye that measures hydroxyl, peroxyl, and other ROS activity within the cell, was added to the cells growing in 96-well plates. After diffusion into the cells, DCFDA was deacetylated by cellular esterase to a non-fluorescent compound, which was then oxidized by ROS into 2′,7′-dichlorofluorescin (DCF), a highly fluorescent compound. The fluorescence from the DCF was detected by fluorescence microplate reader with maximum excitation and emission spectra of 495 and 529 nm, respectively.

**Mitochondrial Membrane Potential Assay**

Mitochondrial Membrane Potential was analyzed using the commercial JC-1 kit (Beyotime, Beijing, China) in accordance with the manufacturer’s protocol. Briefly, HL-1 cells were seeded into a 24-well plate containing glass cover slips. After the specific treatments, the medium was removed and replaced with fresh medium containing 10 µM of 2'-7'-dichlorofluorescein diacetate (DCFH-DA) solution, and the cells were continued to incubate at 37 ℃ for 20 min. The cells were washed 3 times with PBS, and the immunofluorescence was analyzed using a laser scanning confocal microscope (LSM 980, Zeiss, Jena, Germany).

**Figure legends**

**Figure S1.** Characterization of IOPD patients. (A) Genetic pedigree of the family. II-1 is the proband carrying compound heterozygous mutations. (B) Sanger sequencing shows the proband’s compound mutations of c.1822C>T, p.R608X and c.2662G>T, p.E888X in the GAA gene. (C and D) Cardiac hypertrophy of the proband by echocardiography (C) and electrocardiography (D).

**Figure S~~1~~2.** Characterization of the control iPSC from the mother (Ctrl-iPSC). (A) Representative image showing the typical morphology of Ctrl-iPSC. Scale bar, 50μm. (B) Sanger sequencing of the GAA gene containing heterozygous mutation of c.2662G>T, p.E888X in Ctrl-iPSC. (C) Karyotype analysis showing Ctrl-iPSC has normal karyotypes. (D-E) Pluripotent markers of iPSC, NANOG, OCT4, SOX2, and TRA-1-60 (D), and self-renewal marker, Ki67 (E) by IF assay. Scale bar, 50μm. (F) The differentiation of Ctrl-iPSC into three germ layers iPSC(ectoderm, PAX6; endoderm, FOXA2; mesoderm, SOX17) in-vitro trilineage differentiation assay. Scale bar, 50μm.

**~~Figure S2.~~** ~~Identification of the iPSC derived cardiomyocytes. (A) IF showing the expression of cardiomyocytes marker cTNT in Ctrl-iCM and PD-iCM. Scale bars, 20μm. (B-C) Quantitative RT-PCR analysis for cardiomyocytes markers (ACTN2 and TNNT2) from Ctrl-iCM and PD-iCM. Data are presented as “mean ± SD”. *p < 0.05, and **p < 0.01. (Student’s t-test, n = 3).~~

**Figure S3.** Identification of the iPSC derived cardiomyocytes. (A) IF showing the expression of cardiomyocytes marker cTNT in Ctrl-iCM and PD-iCM. Scale bars, 20μm. (B) Quantitative RT-PCR analysis for cardiomyocytes markers (ACTN2 and TNNT2) from Ctrl-iCM and PD-iCM. (C-D) Representative images of flow cytometry (C) and quantitative data (D) from Ctrl-iCM and PD-iCM. (E-G) Quantitative RT-PCR analysis for cardiomyocytes maturity markers, sarcomeric structure (E), calcium handling (F) and iron channels (G). Data are presented as “mean ± SD”. *p < 0.05, and **p < 0.01. (Student’s t-test, n = 3).
